# Rice bran oil could favorably ameliorate atherogenicity and insulin resistance indices among men with coronary artery disease: post hoc analysis of a randomized controlled trial

**DOI:** 10.1186/s12944-021-01584-9

**Published:** 2021-11-06

**Authors:** Marjan Mahdavi-Roshan, Arsalan Salari, Azin Vakilpour, Amir Savar Rakhsh, Zeinab Ghorbani

**Affiliations:** 1 Cardiovascular Diseases Research Center, Department of Cardiology, Heshmat Hospital, School of Medicine, Guilan University of Medical Sciences, 15 Khordad Street, District 2, Rasht, Guilan Province Iran; 2 Department of Clinical Nutrition, School of Medicine, Guilan University of Medical Sciences, Rasht, Iran

**Keywords:** Atherogenic factors, Cardiovascular disorders, Diabetes, Lipoproteins, Rice bran oil

## Abstract

**Background:**

Despite recent advances in recognizing more reliable indicators to estimate the coronary artery disease (CAD) patients’ response to treatment and prognosis, less attention has been paid to evaluating them in clinical trials. Hence, the present research was conducted to study the impact of rice bran oil (RBO) versus sunflower oil (SFO) on various atherogenicity and insulin resistance markers.

**Methods:**

In the present 8-week randomized controlled trial, 40 CAD men with an average age of 56 years were allocated randomly into the intervention or control group to use RBO or SFO (30 g/day) plus a standardized dietary plan. As a further analysis, eight atherosclerosis-related indices were calculated before and after the study.

**Results:**

Analysis of covariance test in which potential confounders and baseline levels were considered, indicated that using RBO compared to SFO reduced Castelli’s risk index I and II (adjusted means:3.29, 1.52 vs. 4.61, 2.20, respectively), atherogenic coefficient (2.29 vs. 3.61), lipoprotein combine index (6.54 vs. 17.53), and cholesterol index (0.46 vs. 1.20) after the trial (*P-value* ≤ 0.002). Also, the RBO group yielded significantly lower triglyceride glucose index (8.73 vs. 9.13) (*P-value* = 0.010). Further, marginally significant amelioration in triglyceride/HDL ratio and atherogenic index of plasma (1.48 and 0.13 vs. 1.86 and 0.24 respectively) were noted (*P-value* = 0.07). Spearman correlation analysis detected significant positive correlations between alterations in TNF-α serum levels (ng/L) and the majority of evaluated indices (*P-value < 0.05*).

**Conclusion:**

Taken together, incorporating 30 g of RBO into the patient’s usual diet appeared effective in ameliorating atherogenicity and insulin resistance indicators among men with CAD, probably in relation to its anti-inflammatory properties.

**Trial registration:**

The protocol of the current trial was retrospectively recorded in the Iranian clinical trial registration system (IRCT) with the registration number of IRCT20190313043045N1 (URL: https://en.irct.ir/trial/38346; Registration date: 2019-04-27).

## Introduction

As a chronic inflammatory condition of vessels, atherosclerosis is known to occur through the growth of lipid-rich lesions, monocytes and macrophages accumulation over the vessels walls inner layer and releasing pro-inflammatory mediators which result in subsequent atheroma formation [1, 2]. It is worth mentioning that males tended to have an augmented burden of severe atherosclerosis events by more than two times as much as females [3]. Conventionally, individualized lipid parameters in the serum, such as triglyceride, high-density lipoproteins-cholesterol (HDL-C), low-density lipoproteins-cholesterol (LDL-C), and total cholesterol, have been well-established as indicators of coronary artery disease (CAD) risk [4–7]. However, they yield limited information and would not be able to accurately reflect the severity of atherosclerotic lesions progression, arterial stiffness, and poor prognosis in CAD patients [5, 7–14]. Given that prompt and proper diagnosis of cardiovascular diseases (CVDs) modifiable risk factors, most importantly dyslipidemia, at early stages could prevent subsequent metabolic disorders, especially CAD, focusing solely on traditional lipid components, including LDL-C as a treatment target, has become a matter of debate [4–7, 10–12, 15]. Accordingly, during recent years, emerging pieces of evidence have proposed novel biomarkers such as lipoprotein(a) and atherosclerosis-related indices [4–8, 11, 16, 17]. These indices which are mainly based on lipoprotein ratios would be able to better predict the risk of CAD, particularly among those having other risk factors. They seem to reflect small dense low-density lipoprotein (sdLDL) or LDL-C particles, and show more robust effects on atherosclerosis development and carotid artery stenoses [4–8, 11]. Although the 2002 National Cholesterol Education Program guideline has mentioned sdLDL measurement could be considered as a complementary treatment target for patients with CAD and chronic metabolic disorders [18]; it, however, cannot be routinely assessed owing to being expensive, time-consuming, and difficult to measure [4–8, 11]. Thus, several attempts have been made to identify surrogate markers of sdLDL and comprehensive atherosclerotic-related indices, including Castelli’s risk indices I and II (CRI-I and CRI-II), lipoprotein combine index (LCI), atherogenic coefficient (AC), triglyceride to HDL-C ratio, triglyceride glucose (TyG) index, atherogenic index of plasma (AIP), and cholesterol index (CHOLINDEX) [19, 20].

On the other hand, considering the renewed interest in incorporating more healthy functional food items into the patients’ usual diet, much concern has been given to the rice bran oil (RBO) health advantages particularly in the past two decades. RBO contains a relatively high load of unsaturated fatty acids (43 and 22% poly- and mono-unsaturated fatty acids (PUFA and MUFA) [21–23]. Besides, a variety of further health advantages of RBO are ascribed to its nutraceutical elements [21, 22, 24, 25]. Nevertheless, limited controlled trials are available concerning RBO impacts on various metabolic disorders. In the light of these pieces of evidence, recently, the current research team has conducted a randomized controlled trial (RCT) aiming to explore the metabolic capabilities of RBO compared to the conventional oil, sunflower oil (SFO), plus a standard diet, among the men suffering from CAD. It was delineated that RBO consumption resulted in improving left ventricular dysfunction and serum levels of metabolic and inflammatory markers and single lipid parameters, except for HDL-C [26]. Considering that the atherogenicity and insulin resistance indices mentioned above could further the present knowledge regarding atherosclerosis progression and yield a deeper understanding of lipoproteins balance, insulin resistance, and response to treatment among CAD patients, in the present research, therefore, it is attempted to extend the previous analysis. Hence, the efficacy of RBO in comparison with SFO on CRI-I, CRI-II, triglyceride to HDL-C ratio, TyG index, AC, AIP, LCI, and CHOLINDEX as novel indicators of metabolic abnormalities in males with coronary disease is explored.

## Methods

### Participants and intervention

The research design and data gathering details have been described elsewhere [26]. Concisely, forty male patients with the diagnosis of severe CAD following percutaneous coronary angioplasty in Heshmat Cardiovascular hospital, Guilan University of Medical Sciences (GUMS), were included in the present RCT from 2019 (April) to 2020 (September). Eligibility criteria, as described in Table 1, required individuals to be male, between 30 and 70 years, diagnosed with CAD, and having BMI less than 35 kg/m2. Patients who had any history of serious cardiovascular events, i.e., surgery, thromboembolism, acute coronary syndrome, cardiogenic shock or coronary artery bypass graft during the 6 months preceding the research, and those who had any comorbidities (i.e., kidney disorders, type 1 diabetes, concurrent viral infections, malignant diseases, or immune system-related disorders) were excluded from the trial. Also, those who had specific dietary habits or consumed dietary supplements during the month before the beginning of the study were excluded. Other exclusion criteria constituted suffering from abuse of alcohol or drugs, any alterations in the therapeutic approaches or medications throughout the trial, and disinclination to participate or keep on the study owing to any reason.

**Table 1:** Eligibility criteria for participants of an 8-week randomized controlled trial comparing the effects of RBO or SFO plus a standard diet on coronary artery disease

Inclusion criteria	Exclusion criteria
Being male	Having any history of serious cardiovascular events (i.e., surgery, thromboembolism, acute coronary syndrome, cardiogenic shock or coronary artery bypass graft) during the 6 months preceding the research
Between 30 and 70 years	Having any comorbidities (i.e., kidney disorders, type 1 diabetes, concurrent viral infections, malignant diseases, or immune system-related disorders)
Diagnosed with CAD	Having specific dietary habits or consumed dietary supplements during the month before the beginning of the study
Having body mass index (BMI) less than 35 kg/m2	Abuse of alcohol or drugs
	Any alterations in the therapeutic approaches or medications throughout the trial
	Disinclination to participate or keep on the study owing to any reason

*RBO* Rice bran oil, *SFO* Sunflower oil

The trial was carried out within 3 days of angioplasty. Of 130 CAD patients the study interventional cardiologists initially examined, 40 individuals who fulfilled the inclusion criteria were assigned to each study arm to take A (RBO) or B (SFO) oil cans in a one-to-one randomization ratio (in blocks of four) using random permuted blocks. The four blocks were generated based on the lists of randomized codes (using the website http://www.randomization.com). Patients in the intervention or control arm were asked to eat 30 g RBO daily or the same amount of SFO, respectively, plus a standardized dietary plan accompanied by routine therapeutic approaches. All enrolled subjects in both RBO or SFO receiving groups were prescribed the same drugs. The RBO used in the present study was a product of Giltaz Company located in Rasht, Guilan province in Iran. The details on the fatty acid composition of the applied oils have been illustrated earlier [26].

The current trial design was confirmed by the Cardiovascular Diseases Research Center, GUMS, with the research number of 97,111,302, and carried out under the Declaration of Helsinki guideline. Also, the ethics committee of GUMS ascertained the study procedures with the ethics number of IR.GUMS.REC.1397.485. The protocol of this trial was retrospectively recorded in the Iranian clinical trial registration system (IRCT) with the registration number of IRCT20190313043045N1 (URL: https://en.irct.ir/trial/38346; Registration date: 2019-04-27). Because of the ethical issues, the usual drug therapies were continued for all studied subjects. All included individuals were provided informed consent in written format.

### Data collection

At the initiation, the data on patients’ demographic, socioeconomic and previous medical history was gathered via an in-person interview. Anthropometric data (i.e., height and weight) were evaluated at the initial visit and after the research. After that, the calculation of body mass index (BMI) was performed by dividing the value of weight (in kg) by height (in square meters). Besides, calculation of energy and macronutrient needs and the prescription of a standard customized healthy diet in accordance with the “United States Department of Agriculture (USDA) food guidelines for Americans, 2010” for all patients were performed by a registered dietitian.

At the first visit and post-intervention, fasting venous blood samples were collected from all enrolled individuals to assess lipid profile, fasting blood sugar (FBS), and serum inflammatory mediators (tumor necrosis factor-alpha (TNF-α) in addition to high-sensitive C-reactive protein (hs-CRP)). More details on laboratory analysis have been reported in the previous publication [26].

### Definition of the atherosclerosis related indices

The following formulas were applied to estimate the atherogenicity and insulin resistance indices in the current post-hoc analysis of RCT:

[10, 19].

$$ \mathrm{Castelli}\ \mathrm{risk}\ \mathrm{index}\hbox{-} \mathrm{I}\left(\mathrm{CRI}-\mathrm{I}\right)=\frac{\mathrm{total}\ \mathrm{cholesterol}\left(\frac{\mathrm{mmol}}{\mathrm{L}}\right)}{\mathrm{HDL}-\mathrm{C}\left(\frac{\mathrm{mmol}}{\mathrm{L}}\right)} $$

[10, 19].

$$ \mathrm{C}\mathrm{astelli}\ \mathrm{risk}\ \mathrm{index}\hbox{-} \mathrm{II}\left(\mathrm{CRI}-\mathrm{II}\right)=\frac{\mathrm{L}\mathrm{DL}\hbox{-} \mathrm{C}\left(\frac{\mathrm{mmol}}{\mathrm{L}}\right)}{\mathrm{HDL}-\mathrm{C}\left(\frac{\mathrm{mmol}}{\mathrm{L}}\right)} $$

[27]

$$ \mathrm{Triglyceride}\ \mathrm{to}\ \mathrm{HDL}\hbox{-} \mathrm{C}\ \mathrm{ratio}=\frac{\mathrm{Triglyceride}\left(\frac{\mathrm{mmol}}{\mathrm{L}}\right)}{\mathrm{HDL}-\mathrm{C}\left(\frac{\mathrm{mmol}}{\mathrm{L}}\right)} $$

[27].

$$ \mathrm{Triglyceride}\ \mathrm{glucose}\ \left(\mathrm{TyG}\right)\ \mathrm{index}= Ln\left(\frac{\mathrm{fasting}\ \mathrm{triglycerides}\ \left(\frac{\mathrm{mg}}{\mathrm{dL}}\right)\times \mathrm{fasting}\ \mathrm{glucose}\ \left(\frac{\mathrm{mg}}{\mathrm{dL}}\right)}{2}\right) $$

[19].

$$ \mathrm{Atherogenic}\ \mathrm{coefficient}\ \left(\mathrm{AC}\right)=\frac{\mathrm{total}\ \mathrm{cholesterol}\left(\frac{\mathrm{mmol}}{\mathrm{L}}\right)\hbox{-} \mathrm{HDL}\hbox{-} \mathrm{C}\left(\frac{\mathrm{mmol}}{\mathrm{L}}\right)}{\mathrm{HDL}\hbox{-} \mathrm{C}\left(\frac{\mathrm{mmol}}{\mathrm{L}}\right)} $$

[10, 19, 20].

$$ \mathrm{Atherogenic}\ \mathrm{index}\ \mathrm{of}\ \mathrm{plasma}\left(\mathrm{AIP}\right)={Log}_{10}\left(\frac{\mathrm{triglycerides}\left(\frac{\mathrm{mmol}}{\mathrm{L}}\right)}{\mathrm{HDL}\hbox{-} \mathrm{C}\left(\frac{\mathrm{mmol}}{\mathrm{L}}\right)}\right) $$

[6, 10].

$$ \mathrm{Lipoprotein}\ \mathrm{combine}\ \mathrm{index}\ \left(\mathrm{LCI}\right)=\frac{\mathrm{total}\ \mathrm{cholesterol}\left(\frac{\mathrm{mmol}}{\mathrm{L}}\right)\times \mathrm{triglycerides}\left(\frac{\mathrm{mmol}}{\mathrm{L}}\right)\times \mathrm{LDL}-\mathrm{C}\ \left(\frac{\mathrm{mmol}}{\mathrm{L}}\right)}{\mathrm{HDL}-\mathrm{C}\ \left(\frac{\mathrm{mmol}}{\mathrm{L}}\right)} $$

[4, 9].

$$ \mathrm{C}\mathrm{holesterol}\ \mathrm{index}\ \left(\mathrm{CHOLINDEX}\right)=\frac{\mathrm{L}\mathrm{DL}\hbox{-} \mathrm{C}\left(\frac{\mathrm{mmol}}{\mathrm{L}}\right)\hbox{-} \mathrm{HDL}\hbox{-} \mathrm{C}\left(\frac{\mathrm{mmol}}{\mathrm{L}}\right)}{\mathrm{HDL}\hbox{-} \mathrm{C}\left(\frac{\mathrm{mmol}}{\mathrm{L}}\right)} $$

(All patients had triglycerides < 400 mg/dL)

### Statistical methods

As it was indicated in previous report [26], the sample size of 20 patients in each study arm was calculated on the basis of considering 80% statistical power (α was set out at 0.05, and β was set out at 0.20) aiming to detect at least 30 mg/dL decrease in total cholesterol concentration with a drop-out rate of 15% (d = 30 and S = 31.58 mg/dL) [28].

For quantitative and categorical variables, the descriptive statistics were provided as mean and standard deviation (SD) or frequencies (%), respectively. After testing for normality applying the Kolmogorov-Smirnov^a^ test, an independent sample t-test or Mann-Whitney U test was done to analyze between-group comparisons of quantitative variables. For categorical data, a chi-square test was conducted. Within-group alterations throughout the trial were also compared applying paired sample t-test or Wilcoxon signed-rank test. For investigating RBO impacts in comparison with SFO on atherogenicity and insulin resistance markers among the studied patients, analysis of covariance (ANCOVA) was run in which age, baseline values, and BMI levels were also considered. Accordingly, means (adjusted) and 95% confidence intervals ​​(95% CI) were provided. Correlation analysis between alterations in TNF-α serum concentration (ng/L) and various indices over the 8 weeks duration of the present trial was explored by applying the Spearman correlation test. Statistical Package for Social Sciences software (version24, SPSS Inc., Chicago, USA) was applied for running statistical analysis. For statistical significance of the performed tests, *P-value* = 0.05 was set out.

## Results

### The participants recruitment procedure and baseline characteristics

The studied subjects’ recruitment procedure has been described elsewhere [26]. Briefly, from the 40 enrolled patients, 37 individuals completed the study. In total, 3 CAD patients were excluded from the RCT; of whom, one patient in the SFO group discontinued the trial due to starting a new dietary plan and two patients in the RBO group failed to complete the study owning to poor adherence to the intervention (*n* = 1) and moving to another city (n = 1). Thus, 18 patients received RBO (mean age = 53.56 years) in addition to a standard diet, and 19 patients consumed SFO (mean age = 57.84 years) plus a standard diet.

Table 2 summarizes the features of the studied patients at the study initiation. The characteristics of both studied arms were comparable in terms of BMI, and age. The means (SD) of BMI of RBO and SFO receiving patients were about 27.00 and 26.08 and kg/m^2^, with no significant differences between groups (Table 2). No significant differences were noted in terms of the distribution of having a history of chronic disorders between the two studied arms. About half of the patients in SFO group and 35% of those in RBO group reported a history of cardiovascular disorders. Besides, approximately half of the subjects in both groups had a history of hypertension. Also, 32 and 10% of the control subjects and 47 and 29% of intervention group showed past medical history of hyperlipidemia and diabetes, respectively. All studied subjects were prescribed routine post angioplasty medications, including aspirin, clopidogrel (Plavix), atorvastatin, losartan, and pantoprazole.

**Table 2:** Baseline characteristics of studied males with coronary artery disease consuming RBO or SFO plus a standard diet in an 8-week randomized controlled trial

	SFO group (***n*** = 19)	RBO group (***n*** = 18)
**Age (year)**	57.84 (7.21)	53.56 (10.99)
**Body mass index (kg/m**^**2**^**)**	26.08 (3.99)	27.00 (3.33)
**Left ventricular ejection fraction (%)**	37.89 (10.45)	43.61 (10.12)

The information in the present table was previously reported in [26] with a slightly different format

*RBO* Rice bran oil, *SFO* Sunflower oil

### Between-group changes in atherogenicity and insulin resistance markers

Detailed results of the current RCT on serum metabolic parameters have been reported previously [26]. Table 3 provides alterations in atherogenicity and insulin resistance markers in males with CAD before and after consumption of RBO or SFO plus a standard diet in an 8-week RCT. The baseline levels of cholesterol-associated indicators, including CRI-I and II, AC, LCI, and CHOLINDEX, did not show significantly different results between the patients in the RBO or SFO groups. When comparing the studied groups regarding these indices after the 8-week trial, significant differences were noted (*P-value ≤* 0.010). The improvements in these markers following RBO consumption became more pronounced after considering the baseline values as well as age and BMI applying the ANCOVA. It was further indicated that RBO consumption resulted in reduced levels of CRI-I (adjusted mean (95%CI) = 3.29 (2.80–3.77)), CRI-II (adjusted mean (95%CI) =1.52 (1.22–1.81)), AC (adjusted mean (95%CI) = 2.29 (1.80–2.77)), LCI (adjusted mean (95%CI) = 6.54 (2.86–10.21)) and CHOLINDEX (adjusted mean (95%CI) = 0.46 (0.15–0.77)) compared to the patients who consumed SFO as the control group (adjusted mean (95%CI) = 4.61 (4.14–5.08), 2.20 (1.92–2.49), 3.61 (3.14–4.08), 17.53 (13.96–21.10), and 1.20 (0.90–1.50), respectively; ANCOVA *P-value* ≤ 0.002) (Table 3).

**Table 3:** Changes in atorvastatin, losartan, and pantoprazoleatherogenicity and insulin resistance indices in males with coronary artery disease before and after consumption of RBO or SFO plus a standard diet in an 8-week randomized controlled trial

	Studied Group		***P-value***
	SFO group (***n*** = 19)	RBO group (***n*** = 18)	
**Castellis risk index-I (CRI-I)**			
Baseline	4.35 (1.32)	4.61 (1.36)	0.554 ^£^
After 8 weeks	4.50 (1.15)	3.42 (1.04)	**0.005** ^£^
Differences	0.14 (1.35)	−1.19 (1.44)	**0.006** ^£^
***P-value*** *	0.646	**0.003**	
Adjusted mean (95%CI)	4.61 (4.14–5.08)	3.29 (2.80–3.77)	**< 0.001** ^δ^
**Castellis risk index-II (CRI-II)**			
Baseline	2.57 (1.22)	2.82 (1.22)	0.529 ^£^
After 8 weeks	2.15 (0.74)	1.58 (0.57)	**0.014** ^£^
Differences	−0.42 (1.18)	−1.24 (1.09)	**0.035** ^£^
***P-value*** *	0.143	**< 0.001**	
Adjusted mean (95%CI)	2.20 (1.92–2.49)	1.52 (1.22–1.81)	**0.002** ^δ^
**Triglyceride to HDL-C ratio**			
Baseline	1.52 (0.85)	2.05 (0.72)	**0.046** ^£^
After 8 weeks	1.73 (0.67)	1.62 (0.65)	0.604 ^£^
Differences	0.21 (0.56)	−0.44 (0.92)	**0.013** ^£^
***P-value*** *	0.112	**0.059**	
Adjusted mean (95%CI)	1.86 (1.57–2.14)	1.48 (1.18–1.77)	0.075 ^δ^
**Triglyceride glucose (TyG) index**			
Baseline	8.95 (0.47)	9.36 (0.45)	**0.010** ^£^
After 8 weeks	9.01 (0.55)	8.87 (0.39)	0.356 ^£^
Differences	0.06 (0.35)	−0.49 (0.47)	**< 0.001** ^£^
***P-value*** *	0.458	**< 0.001**	
Adjusted mean (95%CI)	9.13 (8.94–9.33)	8.73 (8.53–8.93)	**0.010** ^δ^
**Atherogenic coefficient (AC)**			
Baseline	3.35 (1.32)	3.61 (1.36)	0.554 ^£^
After 8 weeks	3.50 (1.15)	2.42 (1.04)	**0.005** ^£^
Differences	0.14 (1.35)	−1.19 (1.44)	**0.006** ^£^
***P-value*** *	0.646	**0.003**	
Adjusted mean (95%CI)	3.61 (3.14–4.08)	2.29 (1.80–2.77)	**< 0.001** ^δ^
**Atherogenic index of plasma (AIP)**			
Baseline	0.12 (0.23)	0.28 (0.17)	**0.019** ^£^
After 8 weeks	0.21 (0.18)	0.17 (0.19)	0.582 ^£^
Differences	0.09 (0.16)	−0.11 (0.23)	**0.005** ^£^
***P-value*** *	**0.034**	**0.058**	
Adjusted mean (95%CI)	0.24 (0.16–0.32)	0.13 (0.05–0.21)	0.076 ^δ^
**Lipoprotein combine index (LCI)**			
Baseline (median (IQR))	12.38 (18.47)	22.32 (27.06)	0.202 ^£^
After 8 weeks (median (IQR))	14.29 (8.83)	7.01 (3.87)	**0.002** ^£^
Differences	−3.43 (14.40)	−17.40 (14.99)	**0.007** ^£^
***P-value*** *	0.376	**< 0.001**	
Adjusted mean (95%CI)	17.53 (13.96–21.10)	6.54 (2.86–10.21)	**< 0.001** ^δ^
**Cholesterol index (CHOLNDEX)**			
Baseline	1.57 (1.02)	1.66 (1.06)	0.789 ^£^
After 8 weeks	1.16 (0.85)	0.51 (0.57)	**0.010** ^£^
Differences	−0.41 (0.93)	−1.16 (0.90)	**0.019** ^£^
***P-value*** *	0.070	**< 0.001**	
Adjusted mean (95%CI)	1.20 (0.90–1.50)	0.46 (0.15–0.77)	**0.002** ^δ^

Data are reported as mean (standard deviation, SD) unless otherwise specified

*RBO* Rice bran oil, *SFO* Sunflower oil, *HDL-C* High density lipoprotein cholesterol

*Paired sample t-test or

^£^Independent sample t-test

^δ^Age, baseline values, and body mass index are considered when running the analysis of covariance (ANCOVA)

In the beginning, the patients in the RBO group had significantly greater triglyceride-related indices (i.e., triglyceride/HDL ratio, TyG index, and AIP) (*P-value* ≤ 0.046). Therefore, although significant within-group reductions in these markers after the 8-week trial were noted among the intervention group, no significant differences were detected based on the independent t-test in the means of triglyceride/HDL ratio, TyG index, and AIP between groups. After adjusting for the potential confounders and the corresponding levels at study baseline in the ANCOVA test, it was revealed that the RBO consuming group exerted a significantly lower TyG index (adjusted mean (95%CI) = 8.73 (8.53–8.93)) than the patients in the SFO receiving group (adjusted mean (95%CI) = 9.13 (8.94–9.33); ANCOVA *P-value* = 0.010). Further, marginally significant ameliorations in triglyceride/HDL ratio (adjusted mean (95%CI) = 1.48 (1.18–1.77)) and AIP (adjusted mean (95%CI) = 0.13 (0.05–0.21)) were observed among the RBO group in comparison with the control patients (adjusted mean (95%CI) = 1.86 (1.57–2.14)) and 0.24 (0.16–0.32), respectively; ANCOVA *P-value* = 0.07) (Table 3).

### Within-group changes in atherogenicity and insulin resistance markers

Regarding within-group changes in comparison with baseline values, the patients who consumed RBO demonstrated statistically significant reductions in the atherogenicity indices, including CRI-I (mean change compared to baseline: − 1.19 (1.44)) and CRI-II (mean change compared to baseline: − 1.24 (1.09)), TyG index (mean change compared to baseline: − 0.49 (0.47)), AC (mean change compared to baseline: − 1.19 (1.44)), LCI (mean change compared to baseline: − 17.40 (14.99)), and CHOLINDEX (mean change compared to baseline: − 1.16 (0.90); *P-value* ≤ 0.003). Besides, marginally significant decreases were noted in the triglyceride/HDL ratio (mean change compared to baseline: − 0.44 (0.92)) and AIP (mean change compared to baseline: − 0.11 (0.23); *P-value* ≤ 0.058) among the patients in the intervention arm; however, no significant alterations were indicated among SFO receiving patients except for AIP which showed a small increase (mean change compared to baseline: + 0.09 (0.16); *P-value* ≤ 0.034).

When comparing the mean changes between the studied groups over the 8-week period, significantly greater declines in CRI-I, CRI-II, triglyceride/HDL ratio, TyG index, AC, AIP, LCI, and CHOLINDEX (*P-value* ≤ 0.035) were detected among the patients in the intervention group (Table 3).

### Correlation analysis between TNF-α levels and atherogenicity and insulin resistance markers

**Fig. 1:**
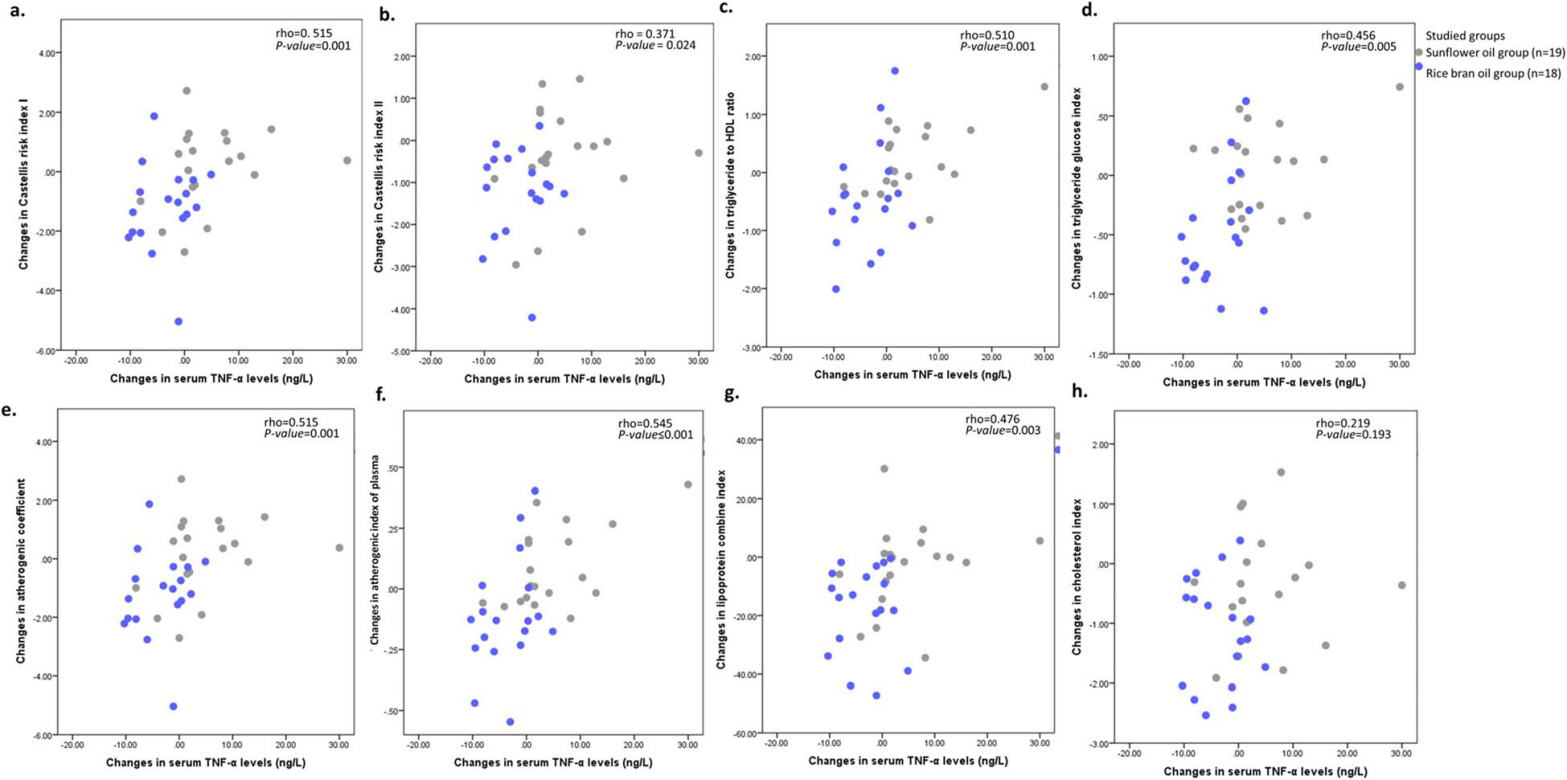
The Correlation between alterations in tumor necrosis factor-α (TNF-α) serum concentration and atherogenic indices levels throughout 8-week trial

As it was observed in the previous report of this trial [26], RBO consumption resulted in attenuating inflammatory status as marked by serum TNF-α diminished levels, while SFO use was accompanied by a slight increment in the levels of TNF-α (ng/L). Accordingly, in the current analysis, the relationship between alterations in TNF-α serum concentration (ng/L) and various atherogenicity and insulin resistance indices before and after consuming RBO or SFO in addition to a standard diet was examined (Fig. 1 (a-h)). Spearman’s correlation analysis yielded significant positive moderate correlations between alterations in CRI-II (rho = 0.371, *P-value* = 0.024), TyG index (rho = 0.456, *P-value* = 0.005) and LCI (r = 0.476, *P-value* = 0.003), and alterations in TNF-α serum concentrations (ng/L) throughout the 8-week trial. Besides, relatively strong and positive correlations were observed between alterations in TNF-α serum concentrations (ng/L) and CRI-I (rho = 0.515, *P-value =* 0.001), triglyceride/HDL ratio (rho = 0.510, *P-value* = 0.001), AC (rho = 0.515, *P-value* = 0.001), and AIP ratio (r = 0.545, *P-value* ≤ 0.001). Moreover, although changes in CHOLINDEX also showed a weak positive correlation with TNF-α alterations (ng/L), this relationship appeared not to be statistically significant.

## Discussion

The present findings underline that incorporating 30 g per day of RBO as the main dietary fat source within a standard diet in comparison with the conventionally consumed oil (SFO) after 8 weeks tended to significantly enhance various atherogenicity and insulin resistance indices.

As far as it is known, no similar clinical trials have been conducted to date. However, the current results, in agreement with the previous findings within the same trial [26], partly corroborate those of prior trials that evaluated the effects of RBO on serum glucose and conventional single lipid parameters and indicated the hypolipidemic and hypoglycemic effects of this oil [28–35]. Notably, the preliminary results of the same trial demonstrated that consuming 30 g RBO per day for 2 months resulted in improving left ventricular dysfunction and serum levels of single lipid parameters (i.e., total cholesterol, triglyceride, and LDL-C) in addition to inflammatory markers, blood sugar, and uric acid [26]. Concordant with these results, a recently published meta-analysis on eight clinical trials with 14 effect sizes found that consuming RBO seems to be effective in alleviating dyslipidemia risk to some extent since it led to enhancing serum levels of lipid components including triglyceride, total cholesterol, and LDL-C. However, as it was previously shown in this trial [26], this oil could not significantly change serum HDL-C levels [36]. Subgroup analysis for BMI also revealed that overweight individuals and those aged > 50 years experienced greater total cholesterol reductions than norm-weight or younger subjects [36]. An earlier meta-analysis on 11 RCT conducted by Jolfaie et al. also revealed similar results [37].

Among various atherosclerosis-related markers, AIP, introduced by Dobiasova et al. [20], can be estimated through the ten logarithmic transformations of the triglycerides to HDL-C ratio. AIP appeared to be well-correlated with LDL-C particles diameters, sdLDL level, and Intima-Media Thickness (IMT). Thus, it could alternatively serve as a strong indicator that well describe the burden of sdLDL cardiovascular complications [6–8, 13, 20, 38–40]. It is noteworthy that sdLDL, as a subcomponent of LDL-C with lower affinity to LDL-C receptors and higher potential for oxidizing and forming foam cells, appeared to be preferred to conventional lipid marker, LDL-C in predicting atherosclerosis risk or prognosis [4–8, 11]. In the clinical setting, the application of AIP as a feasible measure to evaluate the antidyslipidemic potential efficacy of medications for preventing or treating obesity has been gaining wide attention [11, 12, 41]. Moreover, other atherogenicity indicators (i.e., CRI-I and CRI-II, AC, LCI, and CHOLINDEX) that were also observed to be improved following RBO consumption after controlling for baseline levels and confounding factors within the current trial, mainly incorporate the balance between proatherogenic and anti-atherogenic lipid components. In addition, they appeared to be particularly of importance in research on developing novel agents for CVDs treatment [4, 9, 19, 20, 39].

RBO appeared to be an ideal sample of anti-atherogenic functional food with lipid reducing, anti-inflammatory, and anti-oxidant features, as it is not substantially changed during oxidative stress owning to its unsaponifiable fraction and relatively high MUFA content [21, 22, 24, 25]. This heart-friendly oil has been shown to play a favorable role in controlling plasma lipid profile, particularly amongst individuals who have diabetes [21, 42]. Oleic acid makes up 38% of the RBO fatty acid profile, and linoleic and linolenic acids form 34 and 2.2%, respectively [21–23]. Although the exact mechanisms through which RBO could exert anti-hyperlipidemic and anti-hyperglycemic effects are remained to be fully described, its nutraceutical composition can partly explain these beneficial effects. It has been evident that the plant sterols and γ-oryzanol content of RBO in addition to its vitamin E and tocotrienol constituent, which show anti-atherogenic and antioxidant activities, may predominantly contribute to ameliorating hyperlipidemia, hypercholesterolemia, hyperglycemia, hyperinsulinemia, inflammation and lipid peroxidation. Of note, γ-oryzanol constituent of RBO was also indicated to diminish the concentration of plasma non-HDL cholesterol levels, possibly via promoting the release of bile acids and cholesterol in the feces [33, 35, 37, 43–53]. The available evidence indicates that RBO might reduce triglyceride concentration by averting the formation of very low-density lipoprotein cholesterol (VLDL-C) and the lipoproteins that contain apolipoprotein-B100. In addition, RBO could modulate the lipoprotein lipase activity and trigger triglyceride-rich lipoproteins breakdown. In particular, based on the experimental research on hyperlipidemic animals, RBO tocotrienol rich fraction was proved to notably restrain the activity of the main enzyme contributed to the cholesterol metabolism, 3-hydroxy-3-methyl-glutaryl-coenzyme A (HMG-COA) reductase, and through this way, could attenuate hypercholesterolemia in a dose-dependent manner [37, 44, 47, 53]. It has also been mentioned that γ-oryzanol, in addition to tocotrienols, might promote cholesterol 7-alpha-hydroxylase (CYP7A1), the enzyme with rate restriction ability in the synthesis of cholesterol, which stabilizes serum cholesterol concentration by modulating cholesterol-bile acids conversion [33, 37, 53]. With respect to these mechanisms, RBO has also been shown to ameliorate insulin resistance and attenuate lipotoxicity and glucotoxicity [32, 35, 37, 43–46, 48, 54].

Moreover, the TyG index, another proposed indicator of atherosclerosis, is shown to be directly related to lipotoxicity, glucotoxicity, and an increased risk of metabolically unhealthy conditions, including CAD, diabetes, fatty liver arterial stiffness, hypertension, and obesity [7, 27, 55]. In particular, the TyG index that integrates serum fasting concentrations of triglycerides and glucose, has been broadly utilized as a substitute index for exploring resistance to insulin instead of Homeostatic Model Assessment for Insulin Resistance (HOMA-IR) in the medical settings. Proposing a surrogate marker for insulin resistance would be helpful, given that HOMA-IR seems to be costly and time-consuming, and insulin serum concentrations measurement is required to be incorporated in [27, 56, 57]. Concerning this, in the current report, significant reductions in TyG index levels were detected after consuming 30 g of RBO for 8 weeks. Hence, it can be hypothesized that RBO intake could also result in attenuating insulin resistance. The anti-hyperglycemic and insulin resistance improving effects of RBO could also be attributed to its oleic acid content of this oil [32]. Also, the decrease in the liver triglyceride accumulation could play a role in alleviating insulin resistance following RBO, probably owing to its high MUFA content. However, no exact mechanism has yet been proposed for these anti-hyperinsulinemic effects of RBO [53]. From a more detailed perspective, the available experimental research revealed that adding RBO to the diet of rats as the only source of fat seems to decrease HOMA-IR and insulin level, and refine sensitivity to insulin certainly by stimulating the expression of involved genes, including glucose transporters-4 and 5 (GLUT-4 and GLUT-5) in addition to enhancing insulin receptor substrate-1, and insulin receptor activity [58].

Additionally, the positive correlations found between alterations in atherogenicity and insulin resistance markers and TNF-α concentration (ng/L) in the serum were among the interesting findings of the current analysis. These correlations drew our attention to the concept that anti-atherogenic and insulin resistance ameliorating effects of RBO might be partly due to its influences on suppressing inflammation and combating the oxidative stress. As reported in the available evidence, RBO and/or its main components administration seemed to decline the amounts of pro-inflammatory and pro-oxidative mediators (for example, CRP, interleukin (IL)-1b, TNF-α, IL-6, and malondialdehyde). On the flip side, it appeared to elevate the concentration of anti-inflammatory mediators (for example IL-10, IL-4, superoxide dismutase, and catalase) [33, 48–52]. It is noteworthy that RBO and particularly γ-oryzanol and tocotrienol exert antioxidant and anti-inflammatory effects mainly through disturbing nuclear factor-kappa B (NF-κB) p65 pathway and suppressing the release of vascular endothelial growth factors. Besides, these components may play a role in increasing the expression of sterol regulatory peroxisome proliferator-activated receptor gamma (PPARγ) and element-binding protein (SREBP)-2 which could ultimately lead to alleviation of atherosclerosis progression [33, 48–52, 59]. However, the link between atherosclerosis-related markers and inflammatory cytokines as well as the mechanisms by that RBO could favorably affect metabolic and inflammatory status remained to be elucidated more broadly.

### Comparisons with other studies and what does the current work add to the existing knowledge

During previous years, much more consideration has focused on the provision of surrogate markers of sdLDL as well as the suggestion of lipid ratios that comprehensively delineate the balance between lipoproteins with atherogenic and antiatherogenic potentials. These indicators seem to be more practical than traditional single lipid parameters. They could further the present knowledge regarding the early prediction of CAD alongside the effectiveness of the treatment strategies even when other lipid parameters remain within normal ranges [4, 5, 8–11, 38]. However, despite these advances, less attention has been paid to evaluating these indicators in clinical trials exploring the effects of pharmacological or dietary factors on CVDs progression. As far as it is known, there has been no similar RCT in which the changes in atherogenic indices following RBO consumption are investigated. This post hoc analysis of a previously performed RCT, therefore, seems to be the first study to consider these objectives. It was revealed that RBO as the main dietary fat source within a standard diet could significantly enhance atherogenicity and insulin resistance by decreased levels of CRI-I, CRI-II, AC, LCI, CHOLINDEX, and TyG index. RBO also leads to marginally significant enhancements in triglyceride to HDL-C ratio and AIP levels. Additionally, to determine whether RBO could improve the atherogenicity and insulin resistance status through attenuation in inflammation or not, the correlation of alterations in TNF-α serum concentration (ng/L) and various indices over the 8 weeks duration of the trial was examined. Significant correlations were detected between changes in the majority of atherogenicity and insulin resistance markers such as CRI-I, CRI-II, TyG index, AC, LCI, triglyceride to HDL-C ratio, and AIP and serum inflammatory status as measured by TNF-α (ng/L). Hence, it might be speculated that the RBO potential in suppressing inflammation seems to be the key mechanism that contributed to its anti-atherogenic influences. Therefore, encouraging CAD patients to consider this heart-friendly vegetable oil as the main source of daily fat would attenuate atherosclerosis progression and its related metabolic disorders. However, more experimental and clinical trials are required to confirm these results and explore the underlying mechanisms.

### Study strength and limitations

To the best of our knowledge, it is the first time that a controlled trial has been conducted on men with CAD aiming to explore the effects of RBO on serum atherogenicity and insulin resistance indices instead of conventional individualized lipid components. These indicators appear to reflect the balance between lipoproteins, depict the patients’ response to the treatment, and explore CAD prognosis better than the traditional single lipid parameters. Therefore, they would be important in the research on the efficacy of novel interventions for CAD treatment. On the other hand, applying the proposed atherosclerosis-associated indicators as treatment targets in the clinical setting seems to improve the clinicians’ insight into patients’ responses to treatment. Although the present findings seem promising, considering the fact that only male subjects with CAD were included in this RCT, caution must be applied when interpreting the results as the observed effects might not be simply generalized. Moreover, the small sample size and the post-hoc nature of the study are among the other potential limitations of the current findings. Further, considering ethical issues, it was not possible to remove the routine drug therapy of included patients, thereby, the confounding effects of concurrently prescribed medications could not be excluded. However, to minimize the confounding effects of concurrently administered medications, they were prescribed in a similar manner for participants in both studied arms. Besides, regarding other confounders, it was tried to consider the effects of age and BMI as the confounders using ANCOVA though there might be additional confounding variables that can influence RBO effects on outcomes in CAD. Therefore, in the future trials the assessment and adjustment of these factors as well as including female participants should be taken into the account.

## Conclusion

Taken together, incorporating 30 g of RBO to the patient’s usual diet compared to the conventionally consumed oil, SFO, for 2 months appeared effective in improving atherogenicity and insulin resistance. These improvements were indicated by enhanced values of CRI-I and II, AC, LCI, CHOLINDEX, triglyceride to HDL-C ratio, AIP and TyG index following RBO consumption among men with CAD. The anti-atherogenic ameliorating effects of RBO might be partly related to its anti-inflammatory features considering the positive correlations between changes in the indices and serum TNF-a levels (ng/L).

## Data Availability

The analyzed datasets are available from the corresponding author upon rational request.

## Abbreviations

ANCOVA: Analysis of covariance
AC: Atherogenic coefficient
AIP: Atherogenic index of plasma
BMI: Body mass index
CVDs: Cardiovascular disorders
CRI-I: Castelli’s risk Index-I
CRI-II: Castelli’s risk index-II
CAD: Coronary artery disease
CHOLINDEX: Cholesterol index
CYP7A1: Cholesterol 7-alpha-hydroxylase
95% CI: 95% confidence interval
ELIZA: Enzyme-linked immunosorbent assay
FBS: Fasting blood sugar
HDL-C: High-density lipoprotein cholesterol
HOMA-IR: Homeostatic Model Assessment for Insulin Resistance
HMG-COA: 3-hydroxy-3-methyl-glutaryl-coenzyme A
GLUT-4 and GLUT-5: Glucose transporters-4 and 5
GUMS: Guilan University of Medical Sciences
LCI: Lipoprotein combine index
LPL: Lipoprotein lipase
LDL-C: Low-density lipoprotein cholesterol
MUFA: Monounsaturated fatty acid
NF-κB: Nuclear factor-kappa
PPARγ: Peroxisome proliferator-activated receptor-gamma
PUFA: Polyunsaturated fatty acid
RCT: Randomized controlled trial
hs-CRP: Reactive protein
RBO: Rice bran oil
SFA: Saturated fatty acid
sdLDL: Small dense low-density lipoprotein
SD: Standard deviation
SFO: Sunflower oil
SREBP-2: Sterol regulatory element-binding protein
TyG index: Triglyceride glucose
USDA: United States Department of Agriculture
VLDL-C: Very low density lipoprotein cholesterol

## References

[1] Toma I, McCaffrey TA (2012). Transforming growth factor-β and atherosclerosis: interwoven atherogenic and atheroprotective aspects. Cell Tissue Res.

[2] Spinas E, Kritas S, Saggini A, Mobili A, Caraffa A, Antinolfi P, Pantalone A, Tei M, Speziali A, Saggini R (2014). Role of mast cells in atherosclerosis: a classical inflammatory disease. Int J Immunopathol Pharmacol.

[3] Man JJ, Beckman JA, Jaffe IZ (2020). Sex as a biological variable in atherosclerosis. Circ Res.

[4] Olamoyegun MA, Oluyombo R, Asaolu SO (2016). Evaluation of dyslipidemia, lipid ratios, and atherogenic index as cardiovascular risk factors among semi-urban dwellers in Nigeria. Ann Afr Med.

[5] Zhu L, Lu Z, Zhu L, Ouyang X, Yang Y, He W, Feng Y, Yi F, Song Y (2015). Lipoprotein ratios are better than conventional lipid parameters in predicting coronary heart disease in Chinese Han people. Kardiol Pol.

[6] Cai G, Shi G, Xue S, Lu W (2017). The atherogenic index of plasma is a strong and independent predictor for coronary artery disease in the Chinese Han population. Medicine (Baltimore).

[7] Qin Z, Zhou K, Li Y, Cheng W, Wang Z, Wang J, Gao F, Yang L, Xu Y, Wu Y, He H, Zhou Y (2020). The atherogenic index of plasma plays an important role in predicting the prognosis of type 2 diabetic subjects undergoing percutaneous coronary intervention: results from an observational cohort study in China. Cardiovasc Diabetol.

[8] Garg R, Knox N, Prasad S, Zinzuwadia S, Rech MA (2020). The Atherogenic index of plasma is independently associated with symptomatic carotid artery stenosis. J Stroke Cerebrovasc Dis.

[9] Akpınar O, Bozkurt A, Acartürk E, Seydaoğlu G (2013). A new index (CHOLINDEX) in detecting coronary artery disease risk. Anadolu Kardiyol Derg.

[10] Oguntola SO, Hassan MO, Duarte R, Dix-Peek T, Dickens C, Olorunfemi G, Vachiat A, Paget G, Manga P, Naicker S (2018). Atherosclerotic vascular disease and its correlates in stable black south African kidney transplant recipients. Int J Nephrol Renovasc Dis.

[11] Wang L, Chen F, Xiaoqi C, Yujun C, Zijie L (2021). Atherogenic index of plasma is an independent risk factor for coronary artery disease and a higher SYNTAX score. Angiology.

[12] Zhu X, Yu L, Zhou H, Ma Q, Zhou X, Lei T, Hu J, Xu W, Yi N, Lei S (2018). Atherogenic index of plasma is a novel and better biomarker associated with obesity: a population-based cross-sectional study in China. Lipids Health Dis.

[13] Wen J, Zhong Y, Kuang C, Liao J, Chen Z, Yang Q (2017). Lipoprotein ratios are better than conventional lipid parameters in predicting arterial stiffness in young men. J Clin Hypertens (Greenwich, Conn).

[14] Stone NJ, Robinson JG, Lichtenstein AH, Merz CNB, Blum CB, Eckel RH, Goldberg AC, Gordon D, Levy D, Lloyd-Jones DM (2013). ACC/AHA guideline on the treatment of blood cholesterol to reduce atherosclerotic cardiovascular risk in adults. Circulation.

[15] Wu TT, Gao Y, Zheng YY, Ma YT, Xie X (2018). Atherogenic index of plasma (AIP): a novel predictive indicator for the coronary artery disease in postmenopausal women. Lipids Health Dis.

[16] Cesaro A, Schiavo A, Moscarella E, Coletta S, Conte M, Gragnano F, Fimiani F, Monda E, Caiazza M, Limongelli G, D’Erasmo L, Riccio C, Arca M, Calabrò P (2021). Lipoprotein(a): a genetic marker for cardiovascular disease and target for emerging therapies. J Cardiovasc Med.

[17] Gragnano F, Fimiani F, Di Maio M, Cesaro A, Limongelli G, Cattano D, Calabrò P (2019). Impact of lipoprotein(a) levels on recurrent cardiovascular events in patients with premature coronary artery disease. Intern Emerg Med.

[18] National Cholesterol Education Program (NCEP) Expert Panel on Detection, Evaluation, and Treatment of High Blood Cholesterol in Adults (Adult Treatment Panel III) (2002). Third Report of the National Cholesterol Education Program (NCEP) Expert Panel on Detection, Evaluation, and Treatment of High Blood Cholesterol in Adults (Adult Treatment Panel III) final report. Circulation.

[19] Tecer D, Sunar I, Ozdemirel AE, Tural R, Kucuksahin O, Sepici Dincel A, Ataman S (2019). Usefullnes of atherogenic indices and ca-LDL level to predict subclinical atherosclerosis in patients with psoriatic arthritis?. Adv Rheumatol.

[20] Dobiásová M, Frohlich J (2001). The plasma parameter log (TG/HDL-C) as an atherogenic index: correlation with lipoprotein particle size and esterification rate in apoB-lipoprotein-depleted plasma (FER (HDL)). Clin Biochem.

[21] Pal YP, Pratap AP (2017). Rice bran oil: a versatile source for edible and industrial applications. Journal of oleo science.

[22] Bumrungpert A, Chongsuwat R, Phosat C, Butacnum A (2019). Rice bran oil containing gamma-oryzanol improves lipid profiles and antioxidant status in hyperlipidemic subjects: a randomized double-blind controlled trial. J Altern Complement Med.

[23] Sohail M, Rakha A, Butt MS, Iqbal MJ, Rashid S (2017). Rice bran nutraceutics: a comprehensive review. Crit Rev Food Sci Nutr.

[24] Berger A, Rein D, Schäfer A, Monnard I, Gremaud G, Lambelet P, Bertoli C (2005). Similar cholesterol–lowering properties of rice bran oil, with varied γ–oryzanol, in mildly hypercholesterolemic men. Eur J Nutr.

[25] Wilson TA, Nicolosi RJ, Woolfrey B, Kritchevsky D (2007). Rice bran oil and oryzanol reduce plasma lipid and lipoprotein cholesterol concentrations and aortic cholesterol ester accumulation to a greater extent than ferulic acid in hypercholesterolemic hamsters. J Nutr Biochem.

[26] Mahdavi-Roshan M, Salari A, Ghorbani Z, Nikpey Z, Haghighatkhah M, Fakhr Mousavi A, Gholipour M, Pourfarzad A (2021). The effects of rice bran oil on left ventricular systolic function, cardiometabolic risk factors and inflammatory mediators in men with coronary artery disease: a randomized clinical trial. Food Funct.

[27] Yu X, Wang L, Zhang W, Ming J, Jia A, Xu S, Li Q, Ji Q (2019). Fasting triglycerides and glucose index is more suitable for the identification of metabolically unhealthy individuals in the Chinese adult population: a nationwide study. J Diabetes Investig.

[28] Zavoshy R, Noroozi M, Jahanihashemi H (2012). Effect of low calorie diet with rice bran oil on cardiovascular risk factors in hyperlipidemic patients. J Res Med Sci.

[29] Malve H, Kerkar P, Mishra N, Loke S, Rege NN, Marwaha-Jaspal A, Jainani KJ (2010). LDL-cholesterol lowering activity of a blend of rice bran oil and safflower oil (8:2) in patients with hyperlipidaemia: a proof of concept, double blind, controlled, randomised parallel group study. J Indian Med Assoc.

[30] Eady S, Wallace A, Willis J, Scott R, Frampton C (2011). Consumption of a plant sterol-based spread derived from rice bran oil is effective at reducing plasma lipid levels in mildly hypercholesterolaemic individuals. Br J Nutr.

[31] Salar A, Faghih S, Pishdad GR (2016). Rice bran oil and canola oil improve blood lipids compared to sunflower oil in women with type 2 diabetes: a randomized, single-blind, controlled trial. J Clin Lipidol.

[32] Shakib M-C, Gabrial S, Gabrial G (2014). Rice bran oil compared to atorvastatin for treatment of dyslipidemia in patients with type 2 diabetes. Open Access Macedonian Journal of Medical Sciences.

[33] Bumrungpert A, Chongsuwat R, Phosat C, Butacnum A (2019). Rice bran oil containing gamma-Oryzanol improves lipid profiles and antioxidant status in Hyperlipidemic subjects: a randomized double-blind controlled trial. J Altern Complement Med.

[34] Lai M-H, Chen Y-T, Chen Y-Y, Chang J-H, Cheng H-H (2012). Effects of rice bran oil on the blood lipids profiles and insulin resistance in type 2 diabetes patients. J Clin Biochem Nutr.

[35] Berger A, Rein D, Schäfer A, Monnard I, Gremaud G, Lambelet P, Bertoli C (2005). Similar cholesterol-lowering properties of rice bran oil, with varied gamma-oryzanol, in mildly hypercholesterolemic men. Eur J Nutr.

[36] Pourrajab B, Sohouli MH, Amirinejad A, Fatahi S, Găman M-A, Shidfar F. The impact of rice bran oil consumption on the serum lipid profile in adults: a systematic review and meta-analysis of randomized controlled trials. Crit Rev Food Sci Nutr. 2021:1–11. 10.1080/10408398.2021.1895062. 10.1080/10408398.2021.1895062 33715544

[37] Jolfaie NR, Rouhani MH, Surkan PJ, Siassi F, Azadbakht L (2016). Rice bran oil decreases Total and LDL cholesterol in humans: a systematic review and Meta-analysis of randomized controlled clinical trials. Horm Metab Res.

[38] Suleymanoglu M, Rencuzogullari I, Karabag Y, Cagdas M, Yesin M, Gumusdag A, Cap M, Gok M, Yildiz I (2020). The relationship between atherogenic index of plasma and no-reflow in patients with acute ST-segment elevation myocardial infarction who underwent primary percutaneous coronary intervention. Int J Cardiovasc Imaging.

[39] Tecer D, Sunar I, Ozdemirel AE, Tural R, Kucuksahin O, Sepici Dincel A, Ataman S (2019). Usefullnes of atherogenic indices and ca-LDL level to predict subclinical atherosclerosis in patients with psoriatic arthritis?. Adv Rheumatol.

[40] Gentile M, Iannuzzo G, Simeon V, Mattiello A, Rubba F, Panico C, et al. Association between atherogenic index of plasma and carotid intima-media thickness in a cohort of Mediterranean women. Acta Cardiol. 2020:1–6. 10.1080/00015385.2020.1858537. 10.1080/00015385.2020.1858537 33302810

[41] Dobiasova M, Frohlich J, Sedova M, Cheung MC, Brown BG (2011). Cholesterol esterification and atherogenic index of plasma correlate with lipoprotein size and findings on coronary angiography. J Lipid Res.

[42] Jolfaie N, Rouhani M, Surkan P, Siassi F, Azadbakht L (2016). Rice bran oil decreases total and LDL cholesterol in humans: a systematic review and meta-analysis of randomized controlled clinical trials. Horm Metab Res.

[43] Sohail M, Rakha A, Butt MS, Iqbal MJ, Rashid S (2017). Rice bran nutraceutics: a comprehensive review. Crit Rev Food Sci Nutr.

[44] Wilson TA, Nicolosi RJ, Woolfrey B, Kritchevsky D (2007). Rice bran oil and oryzanol reduce plasma lipid and lipoprotein cholesterol concentrations and aortic cholesterol ester accumulation to a greater extent than ferulic acid in hypercholesterolemic hamsters. J Nutr Biochem.

[45] Most MM, Tulley R, Morales S, Lefevre M (2005). Rice bran oil, not fiber, lowers cholesterol in humans. Am J Clin Nutr.

[46] Chou T-W, Ma C-Y, Cheng H-H, Chen Y-Y, Lai M-H (2009). A rice bran oil diet improves lipid abnormalities and suppress hyperinsulinemic responses in rats with streptozotocin/nicotinamide-induced type 2 diabetes. J Clin Biochem Nutr.

[47] Minhajuddin M, Beg ZH, Iqbal J (2005). Hypolipidemic and antioxidant properties of tocotrienol rich fraction isolated from rice bran oil in experimentally induced hyperlipidemic rats. Food Chem Toxicol.

[48] Ahmed MA, Mohamed MA, Rashed LA, Abd Elbast SA, Ahmed EA (2018). Rice bran oil improves insulin resistance by affecting the expression of antioxidants and lipid-regulatory genes. Lipids.

[49] Yalagala PCR, Sugasini D, Ramaprasad TR, Lokesh BR (2017). Minor constituents in Rice bran oil and sesame oil play a significant role in modulating lipid homeostasis and inflammatory markers in rats. J Med Food.

[50] Rao YPC, Sugasini D, Lokesh BR (2016). Dietary gamma oryzanol plays a significant role in the anti-inflammatory activity of rice bran oil by decreasing pro-inflammatory mediators secreted by peritoneal macrophages of rats. Biochem Biophys Res Commun.

[51] Pushpan CK, Shalini V, Sindhu G, Rathnam P, Jayalekshmy A, Helen A: attenuation of atherosclerotic complications by modulating inflammatory responses in hypercholesterolemic rats with dietary Njavara rice bran oil. Biomed Pharmacother. 2016;83:1387–97. 10.1016/j.biopha.2016.08.001. 10.1016/j.biopha.2016.08.001 27583979

[52] Lee S, Yu S, Park HJ, Jung J, Go G-W, Kim W (2019). Rice bran oil ameliorates inflammatory responses by enhancing mitochondrial respiration in murine macrophages. PLoS One.

[53] Chen C-W, Cheng H-H (2006). A Rice bran oil diet increases LDL-receptor and HMG-CoA reductase mRNA expressions and insulin sensitivity in rats with Streptozotocin/nicotinamide-induced type 2 diabetes. J Nutr.

[54] Cheng HH, Ma CY, Chou TW, Chen YY, Lai MH (2010). Gamma-oryzanol ameliorates insulin resistance and hyperlipidemia in rats with streptozotocin/nicotinamide-induced type 2 diabetes. Int J Vitam Nutr Res.

[55] Abbasi F, Reaven GM (2011). Comparison of two methods using plasma triglyceride concentration as a surrogate estimate of insulin action in nondiabetic subjects: triglycerides × glucose versus triglyceride/high-density lipoprotein cholesterol. Metabolism.

[56] Lambrinoudaki I, Kazani MV, Armeni E, Georgiopoulos G, Tampakis K, Rizos D, Augoulea A, Kaparos G, Alexandrou A, Stamatelopoulos K (2018). The TyG index as a marker of subclinical atherosclerosis and arterial stiffness in lean and overweight postmenopausal women. Heart Lung Circ.

[57] Liu XC, He GD, Lo K, Huang YQ, Feng YQ (2020). The triglyceride-glucose index, an insulin resistance marker, was non-linear associated with all-cause and cardiovascular mortality in the general population. Front Cardiovasc Med.

[58] Mohamed MA, Ahmed MA, Abd Elbast SA, Ali NA (2019). Rice bran oil ameliorates hepatic insulin resistance by improving insulin signaling in fructose fed-rats. J Diabetes Metab Disord.

[59] Rigo LA, da Silva CR, de Oliveira SM, Cabreira TN, de Bona da Silva C, Ferreira J, Beck RC (2015). Nanoencapsulation of rice bran oil increases its protective effects against UVB radiation-induced skin injury in mice. Eur J Pharm Biopharm.

